# Central auditory tests reveal deficits in HIV treatment

**DOI:** 10.3389/fpubh.2026.1751685

**Published:** 2026-05-20

**Authors:** Abby K. Kambhampaty, Christopher E. Niemczak, Robert A. Hill, Caitlin G. Howe, Samantha M. Leigh, Jonathan Lichtenstein, Robert M. Roth, Monika Adhikari, Linda Zhang, Abigail M. Fellows, Albert Magohe, Paul Palumbo, Jiang Gui, Enica R. Massawe, Jay C. Buckey

**Affiliations:** 1 Dartmouth College, Hanover, NH, United States; 2 Geisel School of Medicine at Dartmouth, Space Medicine Innovation Lab, Lebanon, NH, United States; 3 Geisel School of Medicine at Dartmouth, Department of Quantitative Biomedical Sciences, Hanover, NH, United States; 4 Dartmouth-Hitchcock Medical Center, Department of Medicine, Lebanon, NH, United States; 5 Department of Biological Sciences, Dartmouth College, Hanover, NH, United States; 6 Geisel School of Medicine at Dartmouth, Department of Epidemiology, Lebanon, NH, United States; 7 Geisel School of Medicine at Dartmouth, Department of Psychiatry, Hanover, NH, United States; 8 Geisel School of Medicine at Dartmouth, The Dartmouth Institute for Health Policy and Clinical Practice, Hanover, NH, United States; 9 Muhimbili University of Health and Allied Sciences, Dar es Salaam, Tanzania

**Keywords:** antiretroviral therapy (ART), body-mass index (BMI), central auditory processing, HIV-associated neurocognitive disorder (HAND), human-immunodeficiency virus (HIV), Tanzania

## Abstract

**Background:**

In Sub-Saharan Africa, where HIV is prevalent and neurocognitive screening tools are limited, central auditory tests (CATs) may provide an accessible way to detect neurocognitive deficits. This study examined the relationship between CAT performance and cognitive performance in people with (PLWH) and without HIV (PLWOH) in Dar es Salaam, Tanzania, with a focus on body mass index (BMI), a potential marker of HIV severity. We hypothesized that low BMI would correlate with more severe HIV and greater CAT and cognitive impairment.

**Methods:**

We analyzed cross-sectional data from two longitudinal studies involving individuals with and without HIV in Tanzania. Participants completed CATs and cognitive assessments. For this analysis, we used data from participants’ most recent assessments. CATs assess the brain’s ability to process sound, correlate with broader cognitive function, and require rapid processing and sustained attention. Sociodemographic, antiretroviral therapy (ART) history, and anthropometric data were collected through questionnaires and clinical records. Linear regression models assessed the associations between BMI, ART regimen, and HIV status on CAT and cognitive performance.

**Results:**

Contrary to our hypothesis, higher BMI was associated with poorer CAT and cognitive performance among participants with HIV. The use of fat-soluble ART drugs was linked to worse outcomes.

**Conclusion:**

Higher body fat may lead to underdosing of fat-soluble ART drugs when fixed-dose combinations are used. These findings suggest that CATs may be sensitive to variation in clinical and treatment-related factors in this population. CATs may represent a feasible, low-resource complement to traditional cognitive assessments for detecting subtle differences in function among individuals with HIV.

## Introduction

HIV-associated neurocognitive disorder (HAND) is a spectrum of neurocognitive complications arising from HIV’s effect on the central nervous system (CNS) (1). Despite widespread access to combination antiretroviral therapy (ART), which has dramatically reduced the incidence of severe HIV-related dementia, approximately 50% of PLWH still experience some form of cognitive impairment (1). Traditional cognitive assessments pose many limitations, as they often require trained neuropsychologists, culturally adapted instruments, and controlled testing environments.

Given these limitations, alternative, accessible tools that can detect early neurological dysfunction among PLWH in resource-limited settings are needed. Central auditory tests (CATs) offer a promising solution. CATs are simple, quick, and do not rely on literacy or high levels of education, making them particularly suitable for low-resource environments (2). Complete batteries with four or five tests are shorter and easier to administer than cognitive assessments, requiring only minimal training (2). Moreover, CATs may help detect early signs of CNS dysfunction and track the effects of therapeutic interventions aimed at preserving cognitive health (2).

The central auditory system encompasses the auditory nerve, brainstem pathways, and auditory cortices, and is responsible for the higher-level processing and interpretation of auditory information (3, 4). Peripheral hearing loss typically results from damage to the outer, middle, or inner ear, impairing the detection of low volume (dB) stimuli. Central auditory processing impairment, on the other hand, involves difficulties in how the brain processes and interprets sounds, particularly in complex listening environments such as noisy backgrounds (5). Importantly, individuals may exhibit central auditory processing deficits even with normal peripheral hearing thresholds, suggesting that central dysfunction can exist independently of measurable hearing loss (6).

Our previous studies have shown that performance on CATs correlates significantly with cognitive function in PLWH in Tanzania (2, 7). These prior longitudinal analyses showed that poorer performance on central auditory tests predicted significantly worse trajectories on multiple cognitive measures independent of age and peripheral hearing status (2). This study aimed to determine whether BMI serves as a risk factor for poorer cognitive and central auditory outcomes in PLWH. To ensure that these outcomes were not confounded by unrelated medical conditions or peripheral hearing loss, participants with clinically significant hearing loss were removed from analysis. BMI has previously emerged as an important factor in the development of HAND, particularly in resource-limited settings (8). In a prior analysis of 761 adults living with HIV, both underweight (BMI < 18·5) and overweight/obese (BMI ≥ 23) participants exhibited significantly higher odds of global neurocognitive impairment compared to their normal-weight counterparts (8, 9). Tuberculosis (TB) also contributes to both HIV progression and cognitive impairment, often compounding existing vulnerabilities in immunocompromised individuals (10). Establishing BMI as a risk factor for neurocognitive and central auditory deficits in this population would offer valuable public health insight into the metabolic dimensions of HIV progression and could support more integrated, accessible approaches to treatment and monitoring. We hypothesised that individuals with lower BMI would experience worse HIV-related outcomes, such as poorer performance on both CATs and traditional cognitive assessments, suggesting increased vulnerability to CNS dysfunction. We hypothesised that these HIV-related outcomes would be exacerbated in individuals with a positive TB history.

## Methods

### Study design and population

Cross-sectional data from our longitudinal study, designed to understand how performance on CATs is correlated with neurocognitive trajectories, were used. A total of 486 adults living with HIV and 213 adults living without HIV were recruited for this longitudinal cohort in Dar es Salaam, Tanzania. 224 children living with HIV and 256 children living without HIV were recruited for a separate pediatric longitudinal cohort. All children were between the ages of three and thirteen at the time of enrolment. Participants attended regular research visits throughout the study: children under 6 years of age attended visits every 6 months, while children aged 6 years and older transitioned to annual visits. For descriptive comparisons between PLWH and PLWOH, continuous variables were compared using two-sample t-tests when normally distributed and using the Mann–Whitney U (Wilcoxon rank-sum) test for skewed variables (BMI, PTA, and CD4 count). Categorical variables were compared using chi-square tests or Fisher’s exact tests when expected cell counts were small. Corresponding *p*-values are reported.

Because anthropometric data (e.g., weight, height) had not been collected regularly before 2023 and follow-up schedules varied, this study used cross-sectional data from each participant’s most recent visit. Pediatric participant visits in this dataset ranged from April 12, 2022 to October 27, 2023, and adult participant visits ranged from June 23, 2022 to October 27, 2023. The research protocol was approved by the Committee for the Protection of Human Subjects of Dartmouth College and the Research Ethics Committee of Muhimbili University of Health and Allied Sciences. All procedures followed the Declaration of Helsinki. Written informed consent was obtained from adults; for pediatric participants, consent was obtained from guardians and assent from the children.

### Data collection

At each visit, participants completed a series of questionnaires and underwent cognitive and auditory testing at the Infectious Disease Center in Dar es Salaam. A sociodemographic questionnaire, adapted from the IMPAACT P1104S Family Demographics and Socioeconomic Questionnaire, collected data on language learning, socioeconomic status, age, parental status, and educational background (10). The health history section included self-reported hearing ability, noise exposure, tinnitus, ear infections, ototoxic medication use (e.g., gentamicin, antimalarials, aspirin, diuretics), and current or past TB and HIV treatment. For participants with HIV, the questionnaire captured current and prior ART regimens, changes over time, adherence, and number of regimens. All data were stored in a REDCap database.

To ensure that cognitive and auditory outcomes were not confounded by unrelated medical conditions or hearing loss, we applied exclusion criteria to both cohorts. Adults were excluded for abnormal tympanometry (*n* = 10), concussion history (*n* = 17), ear drainage (*n* = 36), neurodegenerative disease, psychiatric illness, gentamicin exposure (*n* = 8), or chemotherapy (*n* = 0). Participants with a pure-tone average (PTA; average hearing thresholds at 500–4000 Hz) > 25 dB HL in either ear (*n* = 37) were also excluded. To reduce bias from variable follow-up durations, only data from 2023 onward were included. The final adult sample included 344 adults with HIV and 114 without. Similar criteria were used for the paediatric cohort, with a stricter PTA cutoff of 20 dB HL. After exclusions, the paediatric sample included 178 children living with HIV and 236 without. To minimize confounding from peripheral hearing loss when evaluating central auditory processing, participants with elevated PTA were excluded (adults: PTA > 25 dB HL in either ear; children: PTA > 20 dB HL). This approach is supported by prior work demonstrating that performance on central auditory tests (CATs), which rely on adaptive or threshold-based paradigms, remains largely preserved until peripheral hearing loss becomes moderate to severe, and that mild elevations in audiometric thresholds do not substantially impair CAT performance in either adults or children (11). Excluding individuals with clinically significant peripheral hearing loss allowed us to better isolate central auditory and neurocognitive effects associated with HIV, TB, and nutritional factors, rather than secondary consequences of middle or inner-ear pathology. Importantly, even after exclusion, adults living with HIV demonstrated slightly higher mean PTA values than those without HIV, consistent with prior reports that HIV may subtly affect auditory function without producing overt audiometric hearing loss (11).

### Central auditory tests

For our central auditory processing battery, we focused on speech perception in noise, temporal auditory resolution, and dichotic listening. These domains are critical for real-world listening environments and are commonly disrupted in individuals with neurological, infectious, or developmental conditions (2, 6). For all CATs, the Creare Wireless Noise-Attenuating Automated Hearing Test (WAHTS) system was used to administer the task and record responses (12). Brief descriptions of the central auditory tests used in this study are provided below; detailed protocols and validation of these methods are available in Niemczak et al. (2).

Speech-in-noise abilities were assessed using the Hearing in Noise Test (HINT) and the Triple Digit Test (TDT). The HINT included four conditions: three with noise (from the front, right, or left) and one without noise. In each condition, participants repeated sentences played from the front, with the speech level adaptively adjusted based on response accuracy. Noise was fixed at 65 dB SPL, and each noise condition used 20 unique sentences with a speech-shaped noise masker (2). The speech reception threshold (SRT) was calculated as the average signal-to-noise ratio of presentations after the first four sentences. A composite SRT across the three noise conditions was the primary HINT outcome.

The TDT further assessed speech-in-noise processing using digit triplets (e.g., “two-five-eight”) recorded in Kiswahili and presented against Schroeder-phase masking noise. Participants completed 30 total trials, including six warm-up trials. The task employed an adaptive method, like the HINT, adjusting the SNR after each trial based on participant performance: the target speech level increased by 2·0 dB for incorrect digits and decreased by 0·5 dB for correct digits. The final SRT was calculated from the last seven positive-phase trials and served as the main outcome measure (2, 13).

Temporal auditory processing was assessed using the Gap Detection Threshold Test (GAP), which measures the ability to detect brief silent gaps within four-second white-noise blocks. An adaptive algorithm decreased gap duration after two correct responses and increased it after errors. Participants completed training with auditory and visual feedback before the full test. Gap detection thresholds were calculated using a sigmoidal Hill equation fit to all responses, identifying the 50% detection threshold.

Dichotic listening was evaluated with the Staggered Spondaic Word Test (SSW), where staggered two-syllable spondaic words were presented alternately to each ear. Participants repeated all four words in order; errors in word order were scored as reversals. This task taxed auditory integration and interhemispheric transfer via the corpus callosum, sensitive to central nervous system dysfunction (14). The same central auditory test battery—HINT, TDT, GAP, and SSW—was administered to both adult and pediatric participants. All CATs were delivered using the same automated WAHTS platform with identical stimuli, adaptive algorithms, scoring procedures, and outcome metrics across age groups; only age-appropriate instructions and training trials differed.

### Cognitive tests

In adults, cognitive function was assessed using the Tests of Variables of Attention (TOVA), (15) the Montreal Cognitive Assessment (MoCA), (16) and selected Cogstate subtests (17). In children, the Leiter International Performance Scales–Third Edition (Leiter-3) was used.

The TOVA is a computerised, nonverbal tool measuring attention and visual processing speed with high temporal precision (±1 millisecond) (15). It is independent of language and culture and has been used in low-resource settings (15). We analysed total mean response time and ExGaussian *μ* (a refined metric modelling skewed response times) (15). Poor performance on both TOVA and CATs was considered indicative of broader cognitive deficits. The MoCA is a widely used screening tool for mild cognitive impairment in adults across multiple cognitive domains (16). It assesses visuospatial skills via cube copying and clock drawing, executive function through trail making, verbal abstraction, and word fluency, attention using target detection, serial sevens, and digit span tasks, and memory through learning and delayed recall (16).

The Cogstate battery, validated in PLWH, assesses cognitive domains vulnerable to HIV-related decline (17). We administered the One Card Learning Task (visual recognition memory), Continuous Paired Associate Learning Task (visual associative learning), Groton Maze Learning Test with Delayed Recall (memory retention), and One Back Task (attention, working memory, processing speed). Overall cognitive scores combined errors, latency, and accuracy metrics from these tasks (17).

For the paediatric cohort, cognitive function was assessed using the Leiter-3, a nonverbal tool measuring neurocognitive abilities (14, 24, 25). It evaluates fluid and categorical reasoning, visual identification, and mental sequencing, using gestures for instructions to overcome language and cultural barriers. Children respond via pointing, block placement, or paper tasks. We analysed three composite scores: Nonverbal IQ, Processing Speed, and Nonverbal Attention/Memory (14, 25, 26).

### Statistical analysis

A composite cognitive score was first created for each test metric by standardising into z-scores separately for adult and paediatric cohorts to combine multiple cognitive domains into a single, reliable measure that reduces the risk of type I error and increases statistical power. Age was z-scored separately within the adult and paediatric cohorts to account for fundamentally different age distributions, allowing age to function as a within-cohort covariate rather than a directly comparable unit across cohorts. Accordingly, regression coefficients for age were interpreted only within cohorts and were not compared in magnitude between adults and children. This procedure eliminated running multiple statistical models for each individual test. We used the full sample for normalisation, as many participants living with HIV performed well under modern ART. For all cognitive and auditory measures where higher raw scores indicated better performance, scores were reverse-coded so that higher z-scores consistently reflected poorer performance across all tasks. The composite cognitive score was the mean of all task z-scores. A composite central auditory score was also created by averaging standardised auditory test z-scores. Cognitive and central auditory performance were the primary outcomes in regression models, analysed separately for adults and children. BMI was calculated as weight (kg) divided by height (m^2^) and converted to standardized BMI z-scores separately within the adult and paediatric cohorts. Within each cohort, the mean and standard deviation were derived using only participants without HIV, who served as the local reference population. Individual BMI z-scores were calculated by subtracting the cohort mean and dividing by the corresponding standard deviation. This approach provided a locally relevant reference for BMI while avoiding the use of external standards that may not reflect regional growth patterns, nutritional status, or metabolic context in Tanzanian populations. Fat-soluble antiretroviral drug use was coded as a binary variable (Yes/No) among PLWH, with “Yes” indicating current use of at least one fat-soluble antiretroviral medication (lopinavir, dolutegravir, efavirenz, or ritonavir). PLWH not receiving any fat-soluble antiretroviral medication served as the reference group. Regression models included an interaction term between BMI z-score and HIV status to assess effect modification. Based on exploratory analyses indicating differential BMI–outcome relationships by HIV status, interaction models were treated as the primary analytic models and are presented throughout the Results. Models were run in MATLAB using *fitlm*, with coefficients exported for interpretation, revealing individual and combined effects of BMI, HIV, TB, ART, and age on cognitive outcomes.

## Results

### Demographic characteristics

Table 1 presents demographic and clinical characteristics of adults by HIV status. Participants with HIV were older (45.1 vs. 34.4 years, *p* < 0.001), more likely to report a history of tuberculosis (36.9% vs. 7.0%, p < 0.001), and less likely to be male (25.9% vs. 45.6%, *p* = 0.005).

**Table 1:** Adult cohort demographics by HIV status.

Variable	People living without HIV (*n* = 114)	People living with HIV (*n* = 344)	*p*-value
Continuous variables			
Age (years), mean (SD)	34.4 (11.8)	45.1 (10.6)	<0.001
PTA (dB HL), median (IQR)	11.0 (10.5–18.0)	14.0 (12.25–29.0)	<0.001
Weight (kg), mean (SD)	62.89 (16.37)	64.02 (16.32)	0.523
Height (cm), mean (SD)	161.90 (9.22)	160.27 (8.09)	0.072
Upper Arm Circumference (cm), mean (SD)	29.16 (7.82)	29.34 (7.11)	0.817
Waist Circumference (cm), mean (SD)	87.61 (14.90)	90.92 (14.11)	0.033
BMI (kg/m^2^), median (IQR)	20.54 (18.55–23.62)	21.35 (19.38–24.07)	0.13
CD4 Count (cells/μL), median (IQR)	845 (710–1,005)	780 (620–950)	0.64
CD8 Count (cells/μL), mean (SD)	10.20 (1.03)	12.42 (1.28)	0.189
Categorical variables, *n* (%)			
TB history (positive)	8 (7.0%)	127 (36.9%)	<0.001
Male	52 (45.6%)	89 (25.9%)	0.005
BMI category			
Underweight	23 (20.2%)	42 (12.2%)	0.176
Normal	49 (43.0%)	156 (45.3%)	0.887
Overweight	23 (20.2%)	76 (22.1%)	0.862
Class I obesity	9 (7.9%)	43 (12.5%)	0.357
Class II obesity	7 (6.1%)	17 (4.9%)	1
Class III obesity	3 (2.6%)	10 (2.9%)	1

Continuous variables are presented as mean (standard deviation) or median (interquartile range [IQR]), as appropriate. Group differences were assessed using two-sample t-tests for normally distributed variables and Mann–Whitney U tests for skewed variables (BMI, PTA, and CD4 count). Categorical variables are presented as counts (percentages) and were compared using chi-square or Fisher’s exact tests, as appropriate.

Although both groups had clinically normal peripheral hearing, median pure-tone average (PTA) thresholds were higher among individuals with HIV (14.0 dB HL [IQR: 12.25–29.0] vs. 11.0 dB HL [IQR: 10.5–18.0], *p* < 0.001).

No significant differences were observed in most anthropometric measures, including weight, height, upper arm circumference, or BMI; however, waist circumference was significantly higher among individuals with HIV (*p* = 0.033). Median BMI was similar between groups (HIV-negative: 20.54 [IQR: 18.55–23.62]; HIV-positive: 21.35 [IQR: 19.38–24.07], *p* = 0.13), as was median CD4 count (845 [IQR: 710–1,005] vs. 780 [IQR: 620–950], *p* = 0.64).

### Regression results: central auditory and cognitive composite scores in adult cohort

Regression models demonstrated that the relationship between BMI and both central auditory and cognitive outcomes differed by HIV status, consistent with effect modification. In adults (Table 2), older age (estimate = 0.223, 95% CI: 0.135 to 0.311, *p* < 0.001) and the HIV × BMI interaction (estimate = 0.319, 95% CI: 0.059 to 0.579, *p* = 0.017) were associated with worse central auditory performance. The BMI coefficient represents the association among individuals without HIV, while the interaction term indicates that this association differs among individuals with HIV.

**Table 2:** Linear regression model examining the interaction between BMI z-score and HIV status on central auditory performance in adults.

Term	Estimate	tStat	*p*-value	Adjusted *R*-squared
Regression table: central auditory composite				
Positive TB history	0.057 [−0.104, 0.218]	0.624	0.533	0.194
HIV status	0.128 [−0.065, 0.321]	1.293	0.197	
**Age z score**	**0.223 [0.135, 0.311]**	**4.981**	**<0.001**	
BMI z score	−0.078 [−0.299, 0.143]	−0.661	0.509	
**HIV status X BMI interaction**	**0.319 [0.059, 0.579]**	**2.386**	**0.017**	

This table presents results from a multiple linear regression model assessing associations between BMI z-score, HIV status, age (z-scored), and history of tuberculosis with performance on the central auditory composite score in the adult cohort. All coefficients shown are derived from this single, fully specified interaction model. Positive estimates indicate worse auditory performance. Bold values indicate statistical significance.

**Figure 1:**
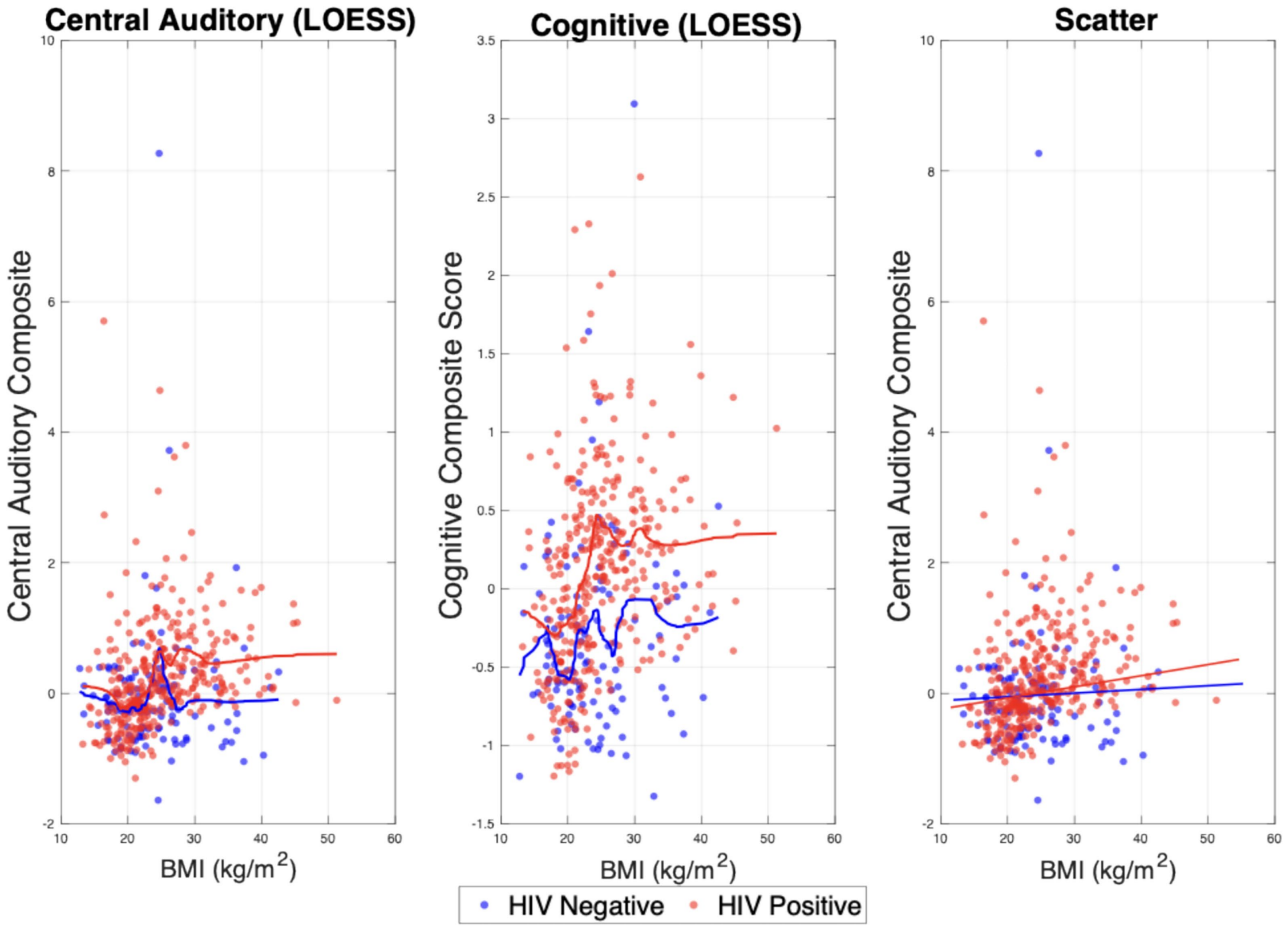
Visualization of central auditory composite score and cognitive composite score vs. BMI in adult cohort. BMI (kg/m^2^) is plotted on the *x*-axis and central auditory composite score on the *y*-axis. Data points for HIV-positive individuals are shown in red and HIV-negative individuals in blue. The first panel displays the LOESS-smoothed relationship for central auditory composite score vs. BMI. The second panel shows the cognitive composite score vs. BMI. The third panel presents the CAT composite score vs. BMI as a scatter plot.

**Figure 2:**
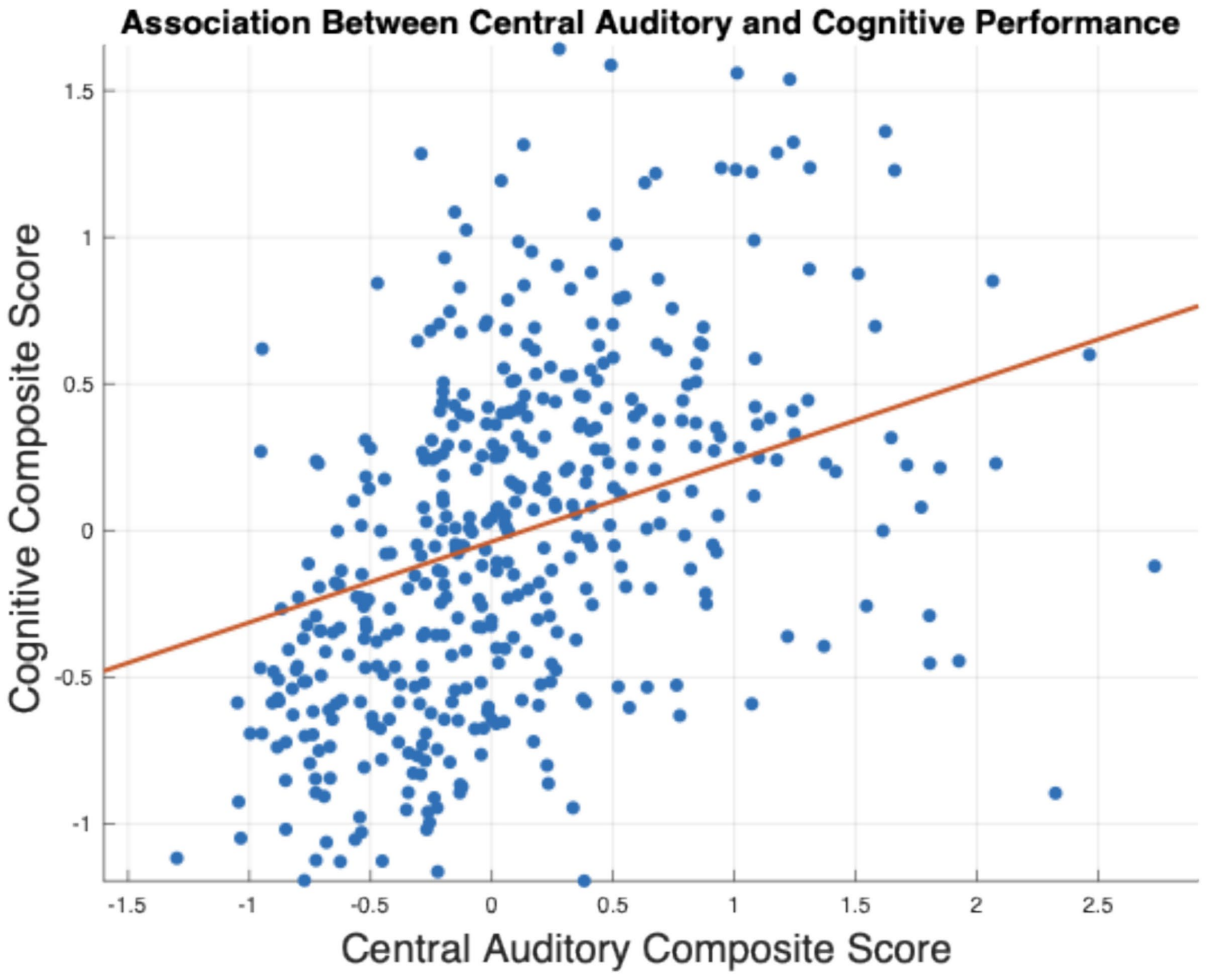
Association between central auditory test performance and cognitive function. Scatterplot illustrating the relationship between central auditory test (CAT) performance and cognitive composite scores across the study cohort.

Cognitive composite models showed similar patterns. In adults (Table 3), HIV status (*p* < 0·001), age (*p* < 0·001), and the HIV·BMI interaction (p = 0·020) were all associated with worse cognitive performance. Relationships between BMI and central auditory in adults are visualized in Figure 1. Central auditory and cognitive composite scores were significantly and positively correlated (*p* < 0.01; *r* = 0.54), as illustrated in Figure 2. Comparable trends were observed in the pediatric cohort, with full results presented in Supplementary Tables S2 and S3.

**Table 3:** Linear regression model examining the interaction between BMI z-score and HIV status on cognitive performance in adults.

Term	Estimate	tStat	*p*-value	Adjusted *R*-squared
Regression table: cognitive composite score				
Positive TB history	0.025 [−0.097, 0.147]	0.420	0.675	0.283
**HIV status**	**0.235 [0.110, 0.360]**	**3.684**	**<0.001**	
**Age z score**	**0.268 [0.210, 0.326]**	**9.279**	**<0.001**	
BMI z score	−0.047 [−0.196, 0.102]	−0.622	0.534	
**HIV Status X BMI interaction**	**0.201 [0.033, 0.369]**	**2.339**	**0.020**	

This table presents results from a multiple linear regression model assessing associations between BMI z-score, HIV status, age (z-scored), and history of tuberculosis with performance on the cognitive composite score in the adult cohort. All coefficients shown are derived from this single, fully specified interaction model. Positive estimates indicate worse cognitive performance. Bold values indicate statistical significance.

Within the PTA-restricted subset, central auditory composite scores remained significantly associated with cognitive composite scores, consistent in both direction and magnitude with our previously published findings. Table 4 summarizes results from adult models testing associations between BMI, fat-soluble ART use (lopinavir, dolutegravir, efavirenz, or ritonavir), and cognitive and auditory outcomes. Use of fat-soluble ARTs was significantly associated with worse cognitive performance (estimate = 0.243, 95% CI: 0.12 to 0.37, *p* < 0.001) showed a non-significant trend toward poorer auditory performance (estimate = 0.160, 95% CI: −0.03 to 0.35, *p* = 0.091). While BMI was not associated with neither cognitive nor auditory outcome for those who were not on fat-soluble drugs, the interaction terms indicate they were associated for those on fat soluble drugs. Comparable pediatric models are presented in Supplementary Table S3.

**Table 4:** Associations between BMI z-score, fat-soluble antiretroviral drug use, and cognitive and central auditory outcomes among adults living with HIV.

Term	Estimate	*t*-Statistic	*p*-value	Adjusted *R*-squared
Model: cognitive composite score				
**Age z-score**	**0.269 [0.21, 0.33]**	**9.409**	**<0.001**	**0.283**
BMI z-score	−0.014 [−0.18, 0.15]	−0.184	0.854	
**Fat-soluble drug (yes)**	**0.243 [0.12, 0.37]**	**3.989**	**<0.001**	
BMI × fat sol drug interaction	0.157 [−0.01, 0.32]	1.853	0.065	
Model: central auditory composite ~ 1 + age_z + BMI_z * fatSolDrug				
**Age z-score**	**0.224 [0.13, 0.32]**	**5.056**	**<0.001**	**0.115**
BMI z-score	−0.052 [−0.29, 0.19]	−0.448	0.655	
Fat-soluble drug (yes)	0.160 [−0.03, 0.35]	1.695	0.091	
**BMI × fat sol drug interaction**	**0.284 [0.03, 0.54]**	**2.159**	**0.031**	

This table presents results from two separate multiple linear regression models conducted among adults living with HIV to assess associations between BMI z-score and neurocognitive or central auditory outcomes, and whether these associations differ by fat-soluble antiretroviral drug use. In these models, the BMI coefficient represents the association between BMI and outcome among participants not receiving fat-soluble drugs, while the interaction term indicates how this association differs among those receiving fat-soluble drugs. Bold values indicate statistical significance.

## Discussion

We hypothesised that low BMI would be associated with poorer central auditory and cognitive outcomes among people living with HIV. In adults (Table 2), age was positively associated with poorer central auditory composite scores. BMI z-score showed a slightly negative association with auditory performance among individuals without HIV, whereas for adults living with HIV, the slope of BMI on outcomes was estimated by the sum of the BMI coefficient plus the HIV × BMI interaction. History of tuberculosis was not independently associated with auditory outcomes. The meaning of age z-scores differs between cohorts; in adults, age spans a broad range and associations with outcomes primarily reflect long-term variability, whereas in the pediatric cohort, the narrower age range implies that even modest age differences may meaningfully influence central auditory and cognitive performance.

Similar patterns were observed for cognitive composite scores in adults (Table 3). BMI z-score was not significantly associated with cognition among HIV-negative individuals. Among adults living with HIV, higher BMI was associated with worse cognitive outcomes. This pattern arises from the significant HIV × BMI interaction term, with HIV status representing a higher intercept and the combined BMI coefficient plus interaction term defining the positive slope observed in the PLWH group. Importantly, these findings reflect effect modification rather than a direct or causal influence of BMI on auditory or cognitive function. As shown in Figure 1, central auditory performance varied as a function of BMI in HIV-positive adults, whereas no comparable association was observed among HIV-negative individuals. Together, these results suggest that metabolic factors may differentially relate to neurocognitive and auditory outcomes in the context of HIV infection. In light of our previous longitudinal work demonstrating that poorer CAT performance predicts adverse cognitive trajectories in people living with HIV (2), the present findings suggest that BMI is associated with both auditory and cognitive outcomes in a manner that differs by HIV status.

In the current study, BMI was not associated with central auditory or cognitive outcomes among adults living without HIV, but was associated with worse outcomes among PLWH, indicating that HIV status acts as an effect modifier of the BMI–outcome relationship. This pattern suggests that metabolic status may be associated with neurocognitive and auditory function in the context of HIV-related immune dysregulation and treatment exposure. BMI has emerged as an important factor in the development of HAND, particularly in resource-limited settings (8, 9, 18, 23). In a prior analysis of 761 adults living with HIV, both underweight (BMI < 18·5) and overweight/obese (BMI ≥ 23) participants exhibited significantly higher odds of global neurocognitive impairment compared to their normal weight counterparts (8, 9). Undernutrition may impair neurodevelopment and immunity, whereas obesity is often linked with comorbidities like hypertension, insulin resistance, and chronic inflammation, all of which are established contributors to cognitive decline (8, 18). This previous study, however, did not address the potential contribution of fat-soluble ART.

### Antiretroviral drugs

This study builds on prior findings by emphasising the role of specific ART drugs, particularly fat-soluble drugs, in shaping both metabolic and neurocognitive outcomes. Efavirenz and lopinavir are highly lipophilic and show altered pharmacokinetics in individuals with obesity (19). In one study, obese patients (median BMI 32·8 kg/m^2^) had significantly lower plasma concentrations of both drugs compared to normal-weight controls (BMI 20–25 kg/m^2^), with identical dosing. Efavirenz levels in obese individuals frequently dropped below its 1 000 ng/mL therapeutic threshold. Similarly, lopinavir levels often fell below its 3 000 ng/mL target, increasing the risk of subtherapeutic exposure.

This is largely due to the drugs’ high lipid solubility, which increases their distribution into adipose tissue and reduces effective plasma levels (19). Importantly, obesity remained a predictor of subtherapeutic levels even after adjusting for confounding variables. These findings underscore the need to consider body composition in dosing, as fixed regimens may be inadequate for individuals with high BMI, potentially increasing the risk of viral rebound and resistance (19).

Ritonavir, another fat-soluble protease inhibitor, is often paired with lopinavir to inhibit CYP3A4 metabolism and enhance efficacy (20). Its pharmacokinetics, like those of lopinavir, are affected by both BMI and dosing. In one study, a reduced 200/50 mg lopinavir/ritonavir dose led to 46%–70% lower plasma concentrations in individuals with high BMI compared to the standard 400/100 mg dose (21). High BMI also affects gastrointestinal absorption and drug metabolism, further decreasing drug bioavailability and reducing effectiveness, warranting monitoring and potential dose adjustments in patients with obesity (20). Dolutegravir, though primarily water soluble, shows moderate fat solubility and slightly reduced exposure in individuals with obesity (22). While the clinical impact is modest and does not require dosing changes, rising obesity rates and weight gain associated with dolutegravir warrant ongoing monitoring. Its lipophilic properties suggest the need for future research into pharmacokinetics in patients with high BMI to guide personalized treatment (12).

### Central auditory and cognitive tests

Previous research from our lab demonstrated strong correlations between central auditory processing and cognitive scores, supporting central auditory tests as indicators of broader neurocognitive function. Our current findings, showing that both HIV status and elevated BMI are linked to poorer central auditory and cognitive performance, reinforce this relationship. These results suggest that central auditory measures are sensitive to both metabolic and infectious disease factors and may serve as practical proxies for neurocognitive health in resource-limited settings. This aligns with growing evidence that sensory processing, especially auditory, is an early marker of neurocognitive decline in paediatric HIV populations (7).

Unlike peripheral hearing tests that assess mechanical sound detection, central auditory tests evaluate neural processing beyond the cochlea (2, 18). After transduction by cochlear hair cells, auditory signals travel through brainstem nuclei, including the cochlear nucleus, superior olivary complex, and inferior colliculus, to the auditory cortex, where features like frequency, timing, and spatial location are encoded. Binaural integration begins at the superior olivary complex, providing the basis for complex sound processing such as speech-in-noise recognition (22).

Our central auditory battery included tasks like speech-in-noise and dichotic listening, which rely on both the ascending auditory pathway and descending cortical feedback circuits (2, 22). Descending fibres from the auditory cortex modulate subcortical processing at sites like the cochlear nucleus and inferior colliculus, enhancing sensitivity to meaningful stimuli (3, 6). These processes engage executive functions such as working memory, processing speed, and attention, which are domains often disrupted by HIV-associated neuroinflammation (1, 4, 10). Central auditory tests are uniquely positioned to advance research and clinical care in low-resource environments. They are inexpensive to administer, do not require extensive infrastructure or high literacy levels, and can be easily adapted to different languages and cultural contexts, making them especially suitable for implementation in sub-Saharan Africa. They can be delivered via tablet or mobile devices using noise-attenuating headphones, allowing testing to occur in schools, clinics, or community settings. Unlike many standardised cognitive assessments, central auditory tests minimise cultural and linguistic bias while still engaging brain systems relevant to learning and development. This accessibility, paired with sensitivity to neurodevelopmental disruptions, makes central auditory tests a promising tool for scalable screening of neurocognitive risk in populations affected by HIV, abnormal BMI, or chronic inflammation.

### Limitations and future directions

This study has several limitations. A key limitation of this study is the absence of longitudinal viral load and CD4 data, which precluded direct assessment of immunologic HIV severity. As a result, BMI was used as a proxy marker, and findings should be interpreted in this context. The CATs, developed with Tanzanian collaborators and used in prior research, lack full formal psychometric validation in Kiswahili, though the HIV-negative group provides a meaningful normative reference. Survey instruments, while not formally validated at the item level, were designed with input from Tanzanian healthcare professionals and community stakeholders for cultural relevance. Analyses of ART regimens were limited by small, uneven drug group sizes, especially for efavirenz, and complex combination therapies, restricting isolation of specific drug effects. Future research should assess drug-dosing effects across the BMI spectrum to identify underexposure thresholds and test whether dose adjustments improve virologic and inflammatory outcomes. Larger, balanced ART subgroup studies are needed to clarify neurocognitive risks of specific drugs, especially efavirenz. Longitudinal studies are essential to understand how early nutrition and infection influence auditory and cognitive development and whether CATs can predict long-term outcomes in HIV and TB populations. Prior studies suggest a non-linear relationship between BMI and neurocognitive outcomes, with increased risk at both low and high BMI ranges; while our analytic approach modeled BMI continuously to preserve power, future studies with larger samples across BMI strata are needed to explicitly test threshold and piecewise effects. The lack of age matching across BMI categories poses an additional limitation, as age is closely related to both body composition and neurocognitive performance and may therefore act as a confounding factor. Although BMI was converted to age-adjusted z-scores and age was included as a covariate in all regression models, residual confounding by age across BMI strata cannot be fully excluded. Another related limitation of this study is the age disparity between PLWH and PLWOH, with the PLWH being significantly older on average. Although age was included as a covariate and z-scored within cohorts in all regression models, age itself is strongly associated with both cognitive and central auditory performance and may partially confound observed group differences. Future studies would benefit from tighter age matching or stratified analyses to more precisely disentangle the independent and interactive contributions of age, HIV infection, and metabolic factors to neurocognitive outcomes.

## Conclusion

Findings from this study suggest that BMI interacts with HIV status to influence central auditory and cognitive outcomes. This interaction is affected by drug regimens, with fat-soluble antiretroviral drugs such as efavirenz, lopinavir, ritonavir, and dolutegravir, suggesting a dual burden of metabolic dysregulation and immune activation. The pharmacokinetic properties of these drugs, particularly in individuals with higher adiposity, may result in subtherapeutic plasma drug concentrations and increase the risk of cognitive impairment. By excluding participants with clinically peripheral hearing loss, our findings specifically reflect central auditory outcomes independent of peripheral auditory hearing loss. The present study suggests that CATs are sensitive to BMI-related changes and represent a low-cost, easily administered tool, particularly useful in resource-limited settings. These findings highlight the need for more personalised ART dosing strategies and support the use of central auditory testing as a frontline screening tool for neurocognitive dysfunction in HIV care.

## Data Availability

The data analyzed in this study is available upon request at any time. Requests to access these datasets should be directed to JB, Jr@dartmouth.edu.
