# Supplementary material for: Central auditory tests reveal deficits in HIV treatment
Source: Front Public Health. 2026 May 20;14:1751685. doi: 10.3389/fpubh.2026.1751685 (PMC13230182; doi:10.3389/fpubh.2026.1751685)
Supplement: Supplementary file 1 [file Supplementary_File_1.docx]

**Supplementary Figures**

*Table 1. Pediatric Cohort Demographics by HIV Status.*

| **Variable** | **People Living without HIV (n = 178)** | **People Living With HIV (n = 236)** | **p-value** |
| --- | --- | --- | --- |
| Continuous variables | | | |
| Age (years), mean (SD) | 8.92 (1.99) | 8.54 (1.89) | 0.05 |
| Weight (kg), mean (SD) | 24.04 (5.90) | 24.70 (8.14) | 0.358 |
| Height (cm), mean (SD) | 125.01 (19.03) | 124.36 (21.11) | 0.749 |
| Waist Circumference (cm), mean (SD) | 58.52 (10.91) | 60.26 (9.51) | 0.085 |
| Upper Arm Circumference (cm), mean (SD) | 20.58 (11.56) | 19.74 (6.69) | 0.354 |
| BMI (kg/m²), median (IQR) | 17.23 (13.2—21.1) | 15.8 (13.0 – 20.5) | 0.41 |
| SES (z-score), mean (SD) | -0.10 (-.6—0.4) | 0.22 (-0.4—0.9) | 0.19 |
| PTA (dB HL), mean (SD) | 15.55 (13.45—17.43) | 15.3 (12.3—17.8) | 0.68 |
| Categorical variables, n (%) | | | |
| TB History (Positive) | 32 (18%) | 2 (1%) | <0.001 |
| Male | 94 (52.8%) | 127 (53.8%) | 0.819 |

**Supplementary Table 1. Pediatric Cohort Demographics by HIV Status.** Continuous variables are presented as mean (standard deviation) or median (interquartile range [IQR]), as appropriate. Group differences were assessed using two-sample t-tests for normally distributed variables and Mann–Whitney U (Wilcoxon rank-sum) tests for skewed variables, where applicable. Categorical variables are presented as counts (percentages) and were compared using chi-square or Fisher’s exact tests, as appropriate.

*Table 2. Linear Regression Model for Pediatric Cohort Cognitive Composite Score with Predictors BMI, HIV, TB and Age*

| **Term** | **Estimate** | **tStat** | **pValue** | **Adjusted R-Squared** |
| --- | --- | --- | --- | --- |
| Positive TB history | 0·226 [−0·078, 0·530] | 1·465 | 0·144 | 0·172 |
| **HIV Status** | **0·401 [0·233, 0·569]** | **4·647** | **<0·001** |  |
| **Age z-score** | **−0·276 [−0·354, −0·198]** | **−6·738** | **<0·001** |  |
| BMI z-score | −0·016 [−0·114, 0·082] | −0·332 | 0·740 |  |
| **HIV Status X**  **BMI interaction** | **0·205 [0·030, 0·380]** | **2·305** | **0·022** |  |

**Supplementary Table 2. Linear Regression Model for Pediatric Cohort Cognitive Composite Score with Predictors BMI, HIV, TB and Age**

This model displays the association between history of TB, BMI z-score, HIV status, and age with performance on a cognitive composite score in our pediatric cohort. The model includes an interaction term between BMI and HIV status.

*Table 3. Linear Regression Model for Pediatric Cohort Central Auditory Composite Score with Predictors BMI, HIV, TB and Age.*

| **Term** | **Estimate** | **tStat** | **pValue** | **Adjusted R-Squared** |
| --- | --- | --- | --- | --- |
| **Positive TB history** | **0·910 [0·152, 1·668]** | **2·366** | **0·018** | **0·337** |
| **HIV Status** | **0·559 [0·154, 0·964]** | **2·704** | **0·007** |  |
| **Age z-score** | **−1·316 [−1·519, −1·113]** | **−13·15** | **<0·001** |  |
| BMI z-score | −0·132 [−0·367, 0·103] | −1·112 | 0·267 |  |
| **HIV Status X BMI interaction** | **0·485 [0·052, 0·918]** | **2·195** | **0·029** |  |

**Supplementary Table 3. Linear Regression Model for Pediatric Cohort Central Auditory Composite Score with Predictors BMI, HIV, TB and Age.**

This model displays the association between history of TB, BMI z-score, HIV status, and age with performance on a central auditory composite score in our pediatric cohort. The model includes an interaction term between BMI and HIV status.

*Table 4. Associations Between BMI, Fat-Soluble Drug Use, and Cognitive and Auditory Outcomes in Pediatric Cohort.*

| Regression Model: Cognitive Composite Score | | | | |
| --- | --- | --- | --- | --- |
| **Variable** | **Estimate** | **tStat** | **pValue** | **Adjusted R-Squared** |
| Positive TB History | 0·234 [−0·07, 0·54] | 1·51 | 0·133 | 0·166 |
| **Age z-score** | **−0·272 [−0·35, −0·19]** | **−6·62** | **<0·001** |  |
| BMI z-score | −0·014 [−0·12, 0·09] | −0·291 | 0·771 |  |
| **Fat-soluble Drug (Yes)** | **0·378 [0·21, 0·54]** | **4·36** | **<0·001** |  |
| **BMI × Fat Sol Drug Interaction** | **0·199 [0·02, 0·38]** | **2·22** | **0·0267** |  |
| Regression Model: Central Auditory Composite | | | | |
| **Variable** | **Estimate** | **tStat** | **pValue** | **Adjusted R-Squared** |
| **Positive TB History** | **0·930 [0·18, 1·68]** | **2·41** | **0·0166** | **0·334** |
| **Age z-score** | **−1·31 [−1·52, −1·10]** | **−13·1** | **<0·001** |  |
| BMI z-score | −0·131 [−0·37, 0·10] | −1·1 | 0·272 |  |
| **Fat-soluble Drug (Yes)** | **0·510 [0·11, 0·91]** | **2·46** | **0·0145** |  |
| **BMI × Fat Sol Drug Interaction** | **0·478 [0·05, 0·91]** | **2·16** | **0·0314** |  |

**Supplementary Table 4. Associations Between BMI, Fat-Soluble Drug Use, and Cognitive and Auditory Outcomes in Pediatric Cohort.** This table summarizes the results of two linear regression models examining the interaction between age (z-scored), BMI (z-scored), and fat-soluble antiretroviral drug use on (1) cognitive composite scores and (2) central auditory composite scores. Fat-soluble drug users include individuals taking Lopinavir, Dolutegravir, Efavirenz, or Ritonavir.
